# Are kinesins required for organelle trafficking in plant cells?

**DOI:** 10.3389/fpls.2012.00170

**Published:** 2012-07-24

**Authors:** Giampiero Cai, Mauro Cresti

**Affiliations:** Dipartimento Scienze Ambientali, University of Siena,Siena, Italy

**Keywords:** kinesin, Golgi, mitochondria, chloroplast, microtubule, organelle movement

## Abstract

Plant cells exhibit active movement of membrane-bounded materials, which is more pronounced in large cells but is also appreciable in medium-sized cells and in tip-growing cells (such as pollen tubes and root hairs). Trafficking of organelles (such as Golgi bodies, endoplasmic reticulum, peroxisomes, and mitochondria) and vesicles is essential for plant cell physiology and allows a more or less homogeneous distribution of the cell content. It is well established that the long-range trafficking of organelles is dependent essentially on the network of actin filaments and is powered by the enzyme activity of myosins. However, some lines of evidence suggest that microtubules and members of the kinesin microtubule-based motor superfamily might have a role in the positioning and/or short-range movement of cell organelles and vesicles. Data collected in different cells (such as trichomes and pollen tubes), in specific stages of the plant cell life cycle (for example, during phragmoplast development) and for different organelle classes (mitochondria, Golgi bodies, and chloroplasts) encourage the hypothesis that microtubule-based motors might play subtle yet unclarified roles in organelle trafficking. In some cases, this function could be carried out in cooperation with actin filaments according to the model of “functional cooperation” by which motors of different families are associated with the organelle surface. Since available data did not provide an unambiguous conclusion with regard to the role of kinesins in organelle transport, here we want to debate such hypothesis.

## ORGANELLE MOVEMENT IN PLANT CELLS

Plant cells are characterized by a powerful flow of intracellular components, generally termed as “cytoplasmic streaming” (Shimmen, 2007). This mechanism allows the efficient transport and the uniform redistribution of membranes and molecules in plant cells, especially in very large cells (Verchot-Lubicz and Goldstein, 2010) such as those of the alga *Chara* that are considered the model par excellence in which to investigate this process (Higashi-Fujime and Nakamura, 2009). It is largely accepted that cytoplasmic streaming is based upon the interaction between actin filaments and myosins (Shimmen, 2007). The *Arabidopsis* genome contains 17 myosin members (Lee and Liu, 2004) and only two plant-specific subfamilies (myosin VIII and myosin XI) have been identified (Shimmen et al., 2000). The rice genome contains 14 myosin genes, with only two of the predicted sequences belonging to class VIII and the others to class XI (Jiang and Ramachandran, 2004). Both myosin XI and myosin VIII subfamilies are closely related to animal and fungal myosin V. Myosins XI are motor proteins showing high processive velocity and are therefore candidates to drive cytoplasmic streaming in plant cells. However, it remains to understand if plant myosins XI interact with specific organelles. Immunolocalization analysis have not clarified this question, since a myosin XI was found to be associated with various particles in tobacco pollen tubes and suspension cells (Yokota et al., 1995). A subclass of myosin XI has been detected in association with mitochondria and plastids (Wang and Pesacreta, 2004) suggesting that it might be used for the transport or positioning of those organelles. By mutant analysis, a specific myosin XI subclass (MYA2) was suggested to play a role in root hair growth and organelle/vesicle trafficking in pollen tubes (Holweg and Nick, 2004; Holweg and Nick, 2008). Analysis of the *Arabidopsis* myosin members showed that myosins MYA1, MYA2, XI-C, XI-E, XI-I, and XI-K were possibly involved in Golgi movement and in mitochondria movement as well (Avisar et al., 2009). Other studies in *Nicotiana benthamiana* also showed that myosin XI-K has a critical role in the trafficking of Golgi bodies, mitochondria, and peroxisomes (Avisar et al., 2008). Comparable results were also obtained for *Arabidopsis*, in which myosins XI-K and XI-2 were found to be responsible for the trafficking of Golgi bodies, peroxisomes, and mitochondria (Peremyslov et al., 2008). In addition, an undetermined form of myosin XI is also responsible for the transport of the endoplasmic reticulum and for its reticular organization (Yokota et al., 2011). These results suggest that plant myosins might exhibit overlapping functions during organelle and vesicle movement in plant cells.

Redistribution of the cell content via cytoplasmic streaming is essential for plant cell growth but this process also requires the local accumulation of specific cell components or even that a given cellular organelle is not part of the streaming. One of the most notable examples is represented by chloroplasts, whose relocation is in most cases independent of cytoplasmic streaming (Liebe and Menzel, 1995). Another example is the endoplasmic reticulum, a membrane network considered as a stationary organelle in quiescent cells while showing movement during induction of streaming (Liebe and Menzel, 1995). Specifically, current hypothesis suggests that the cortical endoplasmic reticulum is relatively static whereas the cytoplasmic reticulum is rapidly streaming by the activity of myosin XI-K (Ueda et al., 2010). Specific changes have been also described for the movement of Golgi stacks, whose transport pattern has been termed as “stop-and-go” because they oscillate rapidly between directed movement and random twisting (Nebenführ et al., 1999). The requirement for a non-uniform distribution of organelles is also exemplified by mitochondria. In pollen tubes, mitochondria continuously move during tube growth according to the reverse flow mechanism, but they are enriched in the subapical region where production of ATP might be more essential (Lovy-Wheeler et al., 2007). Consequently, while cytoplasmic streaming might provide a way to rapidly redistribute organelles in plant cells, an additional (either anchoring or slowing down) phase is also necessary to focus specific organelles into defined cellular areas. While cytoplasmic streaming is driven by the dynamic interplay between actin filaments and myosins, the focusing step might be hypothetically dependent upon microtubules and microtubule-based motors.

## THE MODEL OF FUNCTIONAL COOPERATION

**FIGURE 1:**
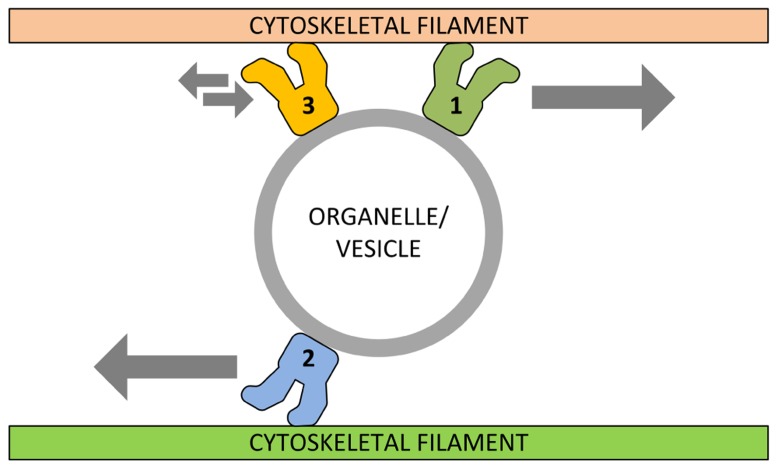
** Schematic model of functional cooperation between different motor proteins.** In its simplest view, specific motor proteins (1) can transport organelles/vesicles along cytoskeleton filaments toward their final destination. Motors of different (or related) families (2) can counteract this activity in order to distribute correctly the organelle content while other motors (3) can even immobilize organelles/vesicles into specific cellular sites.

In animal and fungal cells, transport of organelles is based on the cooperation between different motor proteins and, consequently, on different cytoskeletal systems. The model of “functional cooperation” suggests that a given membrane-bounded structure is specifically delivered to its final destination by the cooperative activity between motors of different families (Goode et al., 2000). The cooperation level might be translated into either a reciprocal assistance for fine-tuning the distribution of organelles or a contrasting action to restrict the delivery sites. For example, depletion of myosin V and VI increased the movement of animal mitochondria as well as their length suggesting that myosins might regulate the movement of mitochondria by opposing the prolonged microtubule-dependent movements in order to facilitate the docking step (Pathak et al., 2010). In fungal cells (hyphae), polarization of cells requires the cooperation of motors of different families, such as kinesins and myosin V, which accumulate in the apex of hyphae, a region enriched in secretory vesicles (Schuchardt et al., 2005). The cooperation between kinesins and myosins is also shown by the transport of cell wall synthetizing enzymes, such as chitin synthase in *Ustilago maydis*. Although actin filaments and microtubules form independent tracks for enzyme delivery, the chitin synthase can travel along both microtubules and actin filaments, a process mediated by kinesin-1 and myosin-5. The final exocytotic step requires the activity of the myosin motor domain, which binds vesicles transiently to actin filaments (Schuster et al., 2012). The myosin-17 motor domain of chitin synthase is also shown to compete with dynein by tethering the chitin synthase to the plasma membrane before exocytosis (Steinberg, 2011). The analysis of relatively simple cell models (such as fungal hyphae and yeast) revealed alternative ways by which motors of different families might cooperate for organelle transport. In budding yeast, kinesin might interact electrostatically with actin filaments preventing the dissociation of cargoes from actin filaments and enhancing the myosin V-based transport of organelles (Hodges et al., 2009). *Xenopus* melanophores are an additional cell model in which the functional cooperation between motors has been investigated; in these cells, pigment organelles (melanosomes) disperse in the cytoplasm or aggregate around the nucleus. Melanosomes are transported by kinesin-2, cytoplasmic dynein, and myosin-V, in a way that is dependent on the number of active motors (e.g., the number of dyneins transporting a melanosome increases during aggregation; Levi et al., 2006). Summarizing these few examples, organelles and vesicles can navigate across the cytoskeletal network in order to reach efficiently their final destination, with motor proteins that reciprocally support each other or even counteract the activity of different motors (**Figure 1**). The rate of organelle transport is likely to depend both on the number of active motors bound to the organelle surface and on the aggregation state of motors (Erickson et al., 2011). Different motors are not only used to travel along different cytoskeletal filaments but they are also required to control the trafficking of organelles through the formation of complex with regulatory proteins, such as protein kinases (Kashina et al., 2004) and Rab-GTPases (Frederick and Shaw, 2007).

## THE KINESIN SUPERFAMILY AND ITS FUNCTION IN PLANT CELLS

The heterogeneous group of kinesins is clustered into the “kinesin superfamily” mainly because of a sequence homology that is often restricted to part of the motor domain (Hirokawa et al., 2009). Kinesins were first identified in the squid giant axon by classical *in vitro* motility assays and by the ATP-dependent co-precipitation with microtubules (Vale et al., 1985). After about 30 years of research, the role, structure and function of kinesins have been progressively elucidated. The motor domain is the feature that distinguishes all members of the kinesin superfamily while the remaining part of molecules varies even consistently and is usually used to determine the specificity of each motor protein and their ability to interact with specific cargoes (Alexander et al., 2009). The kinesin superfamily can be subdivided into several families or groups that have been renamed according to a standardized nomenclature: Kinesin-1 (KHC or kinesin heavy chain), Kinesin-2 (KRP85/95), Kinesin-3 (Unc104/Kif1), Kinesin-4 (Chromokinesin/KIF4), Kinesin-5 (BimC), Kinesin-6 (MKLP1), Kinesin-7 (CENP-E), Kinesin-8 (KIP3), Kinesin-9, Kinesin-10, Kinesin-11, Kinesin-12, Kinesin-13 (MCAK/KIF2), and Kinesin-14 (C-terminal motor; Lawrence et al., 2004). In the plant kingdom, *Arabidopsis thaliana* is the organism for which we have more information. In *Arabidopsis*, not all kinesin subfamilies have been clearly identified and information for specific subfamilies is still very limited (for example, the function of one member putatively belonging to the kinesin-1 subfamily is unknown). Uncertainty also exists for the single member of the kinesin-6 subfamily and for two putative members of the kinesin-8 subfamily. Analysis of identified kinesins suggests that there is no relationship between affiliation to a subfamily and specific function. The kinesin-4 subfamily contains members localized in the cortical microtubule array and putatively involved in regulating the organization of cellulose microfibrils. The kinesin-5 subfamily contains members that might dimerize allowing the sliding of anti-parallel microtubules; since the role of kinesin-5 in spindle structure/function is conserved between organisms, plant kinesins-5 may have evolved additional roles unique to plants. Some members of the abundant kinesin-7 subfamily might be involved in the regulation of phragmoplast assembly as well as in the interaction between chromosomes and microtubules. The kinesin-10 subfamily is likely to be responsible for the delivery of Golgi vesicles in the phragmoplast. The kinesin-12 members are capable of regulating the formation of the preprophase band while the kinesin-13 motor proteins mediate the interaction between Golgi membranes and cortical microtubules. The kinesin-14 subfamily is the most expanded one and contains members with a C-terminal motor domain likely involved in the sliding of adjacent microtubules as it occurs in the formation/functioning of the mitotic spindle and, putatively, in the assembly of the preprophase band. Recently, some plant kinesins have been shown to be unique because they might work as transcriptional activators during gibberellin biosynthesis and cell growth (Li et al., 2012). These examples summarize the concept that plant kinesins might also work differently from their animal counterparts, reflecting the different organization of microtubules in plant cells as well as the specific roles they play. Additional reviews on the genetics of plant kinesins are available (Reddy and Day, 2001, 2011; Lee and Liu, 2004; Richardson et al., 2006; Zhu and Dixit, 2011a).

As a large superfamily, kinesins are involved in several functions in plant cells, both in interphase and dividing cells. In interphase cells, kinesins have been involved in the organization of cortical microtubules. For example, TBK5 (a kinesin-14) was found to be expressed in interphase BY-2 tobacco cells (Matsui et al., 2001) and possibly involved in the transition between cortical and perinuclear microtubules (Goto and Asada, 2007). GhKCBP (a kinesin protein with a calmodulin-binding domain) was found in association with cortical microtubules of cotton fiber cells (Preuss et al., 2003), suggesting the involvement of kinesins in the dynamics of these cytoskeletal structures. The role of kinesins in the organization of cortical microtubules might be even more complex. The kinesin GhKCH1 has a unique calponin homology (CH) domain by which it might bind actin and then mediate the interaction between microtubules and actin filaments during cell growth in cotton fibers (Preuss et al., 2004) and rice (Frey et al., 2009). Some kinesins, like ATK5 (Ambrose et al., 2005), AtKRP125c (Bannigan et al., 2007), and DcKRP120-1 (Barroso et al., 2000) are expressed in interphase cells in addition to dividing cells and they might play a general role in the organization of different microtubule arrays.

Most of plant kinesins are involved in cell division. The preprophase band is likely to be organized by a number of kinesins such as ATK1 (Marcus et al., 2002), KCBP (Bowser and Reddy, 1997), AtKRP125c (Bannigan et al., 2007), and POK1/POK2 (Muller et al., 2006). A prominent role of kinesins is also displayed during the assembly of the mitotic spindle, with kinesins that might interact with both parallel (such as ATK5) and anti-parallel microtubules (AtKRP125c and KCBP) (Vanstraelen et al., 2006). The movement of chromosomes is also dependent on the activity of kinesin-13B and kinesin-7 members. Cytokinesis of plant cells is accomplished by the phragmoplast, a structure formed by two mirrored dynamic microtubule arrays that aligned perpendicularly to the division plane and that served as tracks along which Golgi-derived secretory vesicles move toward the growing cell plate. Members of the kinesin-12 subfamily have been identified in the phragmoplast where they link the plus ends of antiparallel microtubules (Lee et al., 2007); additional kinesins (such as AtPAKRP2) might contribute to the transport of Golgi-derived vesicles in the phragmoplast and thus to the development of the cell plate (Lee et al., 2001).

## WHAT DO WE KNOW ABOUT MICROTUBULE-BASED MOTILITY IN PLANT CELLS?

The involvement of plant microtubules in organelle transport is subtle and often difficult to decipher. Apart from the role of microtubules in chromosome movement, transport of organelle and vesicles along microtubules is documented only by sporadic reports and is limited to few cell types where membrane movement can be studied more easily. The directional grain-to-apex (and vice versa) movement of organelles, accomplished to the focusing of Golgi vesicles in the tip, makes the pollen tube an excellent system where to investigate the contribution of microtubules and microtubule-based motors to organelle trafficking (Derksen, 1996; Cai and Cresti, 2009). Consequently, it is not surprising that the pollen tube is the first plant cell in which kinesins were identified exactly 20 years ago (Tiezzi et al., 1992). Cotton fibers are single-celled trichomes that synchronously undertake a rapid phase of cell expansion, then a phase of secondary cell wall deposition, followed by a final phase of maturation. Since expansion and cell wall deposition require the trafficking of organelles along the cytoskeleton, cotton fibers are excellent models for analyzing the contribution of kinesin-based transport to cell wall deposition (Xu et al., 2009). An additional example of model system is the phragmoplast, a cell structure that is used for cytokinesis of dividing cells. The function of phragmoplast microtubules is to deliver vesicles to the division plane while fusion of vesicles generates the cell plate (Jürgens, 2005). Microtubules are likely involved in the direct transport of vesicles toward the center of phragmoplast and this function is supported by phragmoplast kinesins (Lee and Liu, 2000). In addition to Golgi vesicles, involvement of microtubules in either the transport or positioning of plant organelles has been documented for mitochondria (Van Gestel et al., 2002), Golgi stacks (Crowell et al., 2009), and endoplasmic reticulum (Foissner et al., 2009). Specific inhibitors are often used as tools to determine the role played by cellular structures. Early and more recent studies employing either colchicine or oryzalin (the two most frequently used microtubule inhibitors) suggested that microtubules are not directly involved in cytoplasmic streaming in plants (Bradley, 1973; Lovy-Wheeler et al., 2007). Conversely, the requirement for high-speed movement of organelles (necessary for the efficient distribution of membranes and molecules) resulted in the expansion of the myosin family and therefore in the almost exclusive use of such motors for long-range transport of plant organelles (Vale, 2003). Consequently, most of microtubule-based motors are specialized in the division of plant cells and only few members are likely specifically involved in organelle transport (Reddy and Day, 2001; Lee and Liu, 2004; Vanstraelen et al., 2006; Zhu and Dixit, 2011a).

## EXAMPLES OF KINESIN-BASED MOTILITY OF PLANT ORGANELLES

### MITOCHONDRIA

The interaction of plant kinesins with mitochondria is documented but a general view of the interaction’s function is still unclear. Available information is suggestive but still either controversial or difficult to understand. For example, two plant kinesin-related proteins, MKRP1 and MKRP2 (for mitochondria-targeted KRP), were supposed to work inside *Arabidopsis* mitochondria. Those proteins contain an N-terminal mitochondrial targeting signal and might be a specific subclass of kinesins whose function is currently unknown (Itoh et al., 2001). More generally, plant kinesins were shown to be possibly involved in the dynamic interaction between mitochondria and microtubules. The AtKP1 kinesin-14 has been localized on organelles of either interphase or dividing cells. Although not exclusively, AtKP1 was found to be tightly associated with mitochondria from which it was not removed even by treatment with 0.6 M KI (Ni et al., 2005). This suggests that the protein is normally associated with mitochondria and that the interaction with microtubules is sporadic and possibly required for the regulation of mitochondria positioning. Surprisingly, the protein has been shown to interact with a mitochondrial outer membrane protein, the voltage-dependent anion channel 3 (VDAC3; Yang et al., 2011). Binding of the motor protein to mitochondria is strictly dependent on the presence of VDAC3 and regulates both oxygen consumption and the ratio between the cytochrome pathway and the alternative oxidase pathway (at least at low temperature). Whether the interaction between AtKP1 and VDAC3 also affects the binding of mitochondria to microtubules is not clear. Hypothetically, the kinesin-based positioning of mitochondria might be required to synchronize the respiratory activity with the position of mitochondria; in this view, AtKP1 might be necessary first for establishing contact with microtubules, then for positioning of mitochondria at specific sites and finally for activating the respiration process.

Other results support the hypothesis that kinesins are required for the positioning of mitochondria in plant cells. In cultured cells of tobacco, mitochondria either move along cytoplasmic strands or are steady in the cortical layer. Although active movement is dependent on actin filaments, the microtubule-depolymerizing drug, oryzalin, inhibited the distribution of mitochondria in parallel arrays (Van Gestel et al., 2002). This report does not specifically describe the involvement of kinesins in mitochondria positioning and does not preclude a role for structural MAPs; however, a putative role of such motors in positioning/anchoring mitochondria is fascinating. In the cortex of elongating characean internodal cells, small mitochondria moved slowly along microtubules or actin filaments while the shape and motility of larger mitochondria is based on the interaction with both cytoskeletal filaments. When cells cease to elongate, no microtubule-dependent motility was observed (Foissner, 2004). These observations suggest that mitochondria of characean internodal cells use both microtubule- and actin filament-based motors for either movement or immobilization. Further support to this model came from studies in the pollen tube where three kind of evidence were collected. First, a motor protein biochemically related to kinesins was found in association with membrane structures located in the cortex but not in the tip region (where mitochondria are usually excluded; Cai et al., 2000). Second, organelles isolated from pollen tubes were shown to move actively along taxol-stabilized microtubules in the absence of cytosolic proteins. Most of those organelles grouped into a fraction enriched in cytochrome *c* oxidase activity (a mitochondrial marker) and a protein isolated from the organelle fraction exhibited immunological and biochemical properties of the kinesin family (Romagnoli et al., 2003). Third, mitochondria purified from tobacco pollen tubes were shown to move along both microtubules and actin filaments but with relatively different speeds. While mitochondria moved at 0.17 μm/s along microtubules, they moved more rapidly (at 1.73 μm/s) along actin filaments and at speed of 0.53 μm/s when actin filaments and microtubules were assayed together. Mitochondria showed a rapid, often saltatory, movement along actin filaments but a linear, slow movement along microtubules and they shifted from actin filaments to microtubules. Both kinesin and myosin might be associated with pollen tube mitochondria, as also confirmed by immunolocalization experiments (Romagnoli et al., 2007). Unfortunately, the 90-kDa kinesin-like proteins of pollen tube mitochondria was not characterized at genetic level. A different interpretation of the role played by microtubules in mitochondria positioning has been proposed for the pollen tube of the gymnosperm *Picea wilsonii*. In such case, microtubules are suggested to affect the trajectories of mitochondrial movement rather than their speed by changing the arrangement of actin filaments (Zheng et al., 2010). Although the hypothesis might be plausible, the gymnosperm pollen tube is rather different from the angiosperm one in terms of structure and growth mechanism and a direct comparison is not appropriate.

### GOLGI BODIES

**FIGURE 2:**
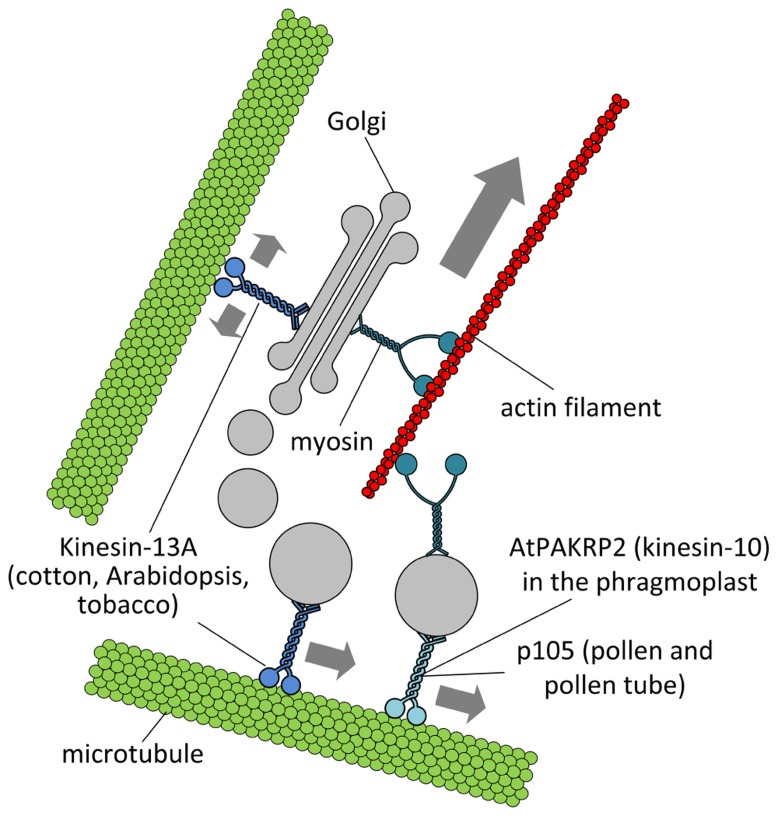
** Putative interactions between Golgi stacks/vesicles and motor proteins.** Specific subclasses of myosin XI would be responsible for the long-range transport of Golgi bodies within plant cells. Members of the kinesin superfamily are likely involved in either the anchorage of Golgi stacks to microtubules (such as kinesin-13A) or the short-range transport of Golgi vesicles to defined cell positions (such as AtPAKRP2 and p105). The arrow length indicates the relative velocity as produced by motor proteins. Opposing arrows indicate an anchorage role for the motor.

In plant cells, the Golgi complex is a motile membranous structure trafficking along actin filaments and that picks up vesicle-delivered products from the stationary endoplasmic reticulum (Boevink et al., 1998; Nebenführ and Staehelin, 2001; Hanton et al., 2005). Golgi stacks are suggested to carry out a so-called “stop-and-go” movement because they oscillate rapidly between directed movement and random twisting. The directed movement is dependent on actin filaments and myosin motors. Specifically, the analysis of annotated *Arabidopsis* myosins for their involvement in Golgi motility has shown that myosins MYA1, MYA2, XI-C, XI-E, XI-I, and XI-K were the best candidates for Golgi movement, although other myosins were also associated with Golgi bodies and some of them also control mitochondria movement (Avisar et al., 2009). On the other hand, microtubules might play a critical role in the “stop” phase of Golgi trafficking (Nebenführ et al., 1999), a process that likely corresponds to a pause along the endoplasmic reticulum and to the capture of vesicle-enclosed products. Since treatment with oryzalin enhances Golgi motility, it was suggested that microtubules might regulate the positioning of Golgi bodies. Currently, no kinesins are shown to take part in the membrane trafficking between ER and Golgi, but the kinesin AtPAKRP2 (a kinesin-10 member) likely contributes to the transport of Golgi-derived vesicles in the phragmoplast (Lee et al., 2001) providing evidence for kinesin–Golgi membrane interactions. Association of kinesin-like proteins with Golgi-derived membranes was also suggested by a pioneering study on *Corylus avellana* pollen. Using biochemical and immunological approaches, a 105-kDa kinesin-like protein was identified in a Golgi vesicle-enriched fraction and localized on the outer surface of Golgi-derived membranes (Liu et al., 1994). Comparable approaches also indicated that a kinesin-like protein might be associated with membranes in the apex of tobacco pollen tubes, a region enriched in Golgi-derived vesicles (Tiezzi et al., 1992; Cai et al., 1993). Genetic evidence for Golgi-associated kinesins was later obtained in *Gossypium hirsutum* and *Arabidopsis thaliana*, where an internal motor kinesin-13A was identified. The protein was localized to entire Golgi stacks, which were regularly associated with microtubules and actin filaments. Since clustering of Golgi stacks was observed in kinesin-13a mutant trichomes, the distribution of Golgi stacks might require microtubules and the kinesin-13A (Lu et al., 2005). These data implied that kinesin-13A might be a candidate for the pausing of Golgi bodies along microtubules. A parallel study with degenerate primers allowed the cloning of a tobacco sequence highly homologous to the kinesin-13A from *Arabidopsis*. The corresponding protein (NtKinesin-13A) was immunolocalized in the tobacco pollen tube and shown to partially overlap with the 58K protein (a marker of Golgi apparatus). However, the motor protein was not found in the pollen tube apex suggesting that it was not critical for the delivery of secretory vesicles (Wei et al., 2005). The functional significance of Golgi pausing along cortical microtubules in plant cells is not clear but recent reports indicate that the pausing step might be important at least for the insertion of the cellulose synthase complex (CSC) in the plasma membrane (Crowell et al., 2009). According to this model, cortical microtubules regulate the insertion of CSCs and guide their movement in the plasma membrane, thus establishing a link between microtubules, CSCs, and, possibly, kinesins. This hypothesis is further discussed in next paragraphs. Immunocytological analysis also showed that AtKinesin-13A co-localized with Golgi stacks and Golgi-associated/derived vesicles in *Arabidopsis* root tip cells, suggesting that AtKinesin-13A might be involved in the formation and/or movement of Golgi vesicles in a variety of plant cells (Wei et al., 2009). A summary of the interactions between kinesins and Golgi membranes is depicted in **Figure 2**.

Whether other microtubule-based motors are required for the localization of Golgi stacks is unknown but intriguing in light of recent findings. Although the presence of dynein-like proteins in plant cells is debated (Wickstead and Gull, 2007), immunological analysis with antibodies to fragments of both microtubule-binding and motor domains of the dynein heavy chain from *Dictyostelium* revealed a polypeptide of high molecular weight (>500-kDa) in root meristem cells of wheat. The antibodies localized the antigen in association with aggregates also labeled by Golgi markers (Shanina et al., 2009). Documentation of putatively dynein-like proteins evokes the identification of dynein polypeptides in tobacco pollen tubes, which were also found in association with membrane structures (Moscatelli et al., 1995). Although these findings are potentially interesting, the absence of genetic information on plant dyneins does not allow to formulate a convincing hypothesis on the role of such proteins during the life cycle of plant cells.

### CHLOROPLASTS

The movement of chloroplasts is quite different from that of other organelles as is dictated by light (photorelocation) and chloroplasts tend to accumulate in the cortical area of plant cells, exhibiting a movement that is neither linear nor continuous. Unlike other organelles, the movement of chloroplasts is not dependent on the interaction between myosin XI and actin filaments but on the polymerization state of short actin filaments (cp-AFs) associated with the outer envelope of chloroplasts (Suetsugu et al., 2010). However, two kinesin-like proteins, KAC1 and KAC2, have been shown to be essential for chloroplast movement and for anchoring of chloroplasts to the plasma membrane. The motor domain of KAC is similar to those of kinesins-14 but both KAC1 and KAC2 do not have microtubule-binding activity. Since the C-terminal domain of KAC1 might interact with actin filaments, KAC proteins might mediate the movement of chloroplasts by interacting with actin filaments (Suetsugu et al., 2010) in a way that would be unique to plants.

### CELL WALL DEPOSITION

The process of cell wall deposition is composed of a series of events starting from the intracellular production of key metabolites (such as UDP glucose) and ending with the synthesis of cellulose microfibrils and the correct assembly of the wall. Historically, microtubules have always been involved in the process of cell wall assembly since first observations of cortical microtubules associated with the plasma membranes (Ledbetter and Porter, 1963). The precise role of microtubules in the cell wall deposition always remained elusive until the movement of cellulose synthase (CesA) was observed *in vivo* (Paredez et al., 2006). This approach showed that CesA could move in the plane of the plasma membrane along trajectories coincident with the pattern of microtubules underlying the plasma membrane. Since depolymerization of microtubules by oryzalin only alter the initial trajectory of movement of CESA complexes (Paredez et al., 2006), the precise interaction between CesA and microtubules is not yet fully clear. The recent identification of proteins forming a complex with CesA and microtubules suggests that microtubules could directly control the positioning and movement of CesA in the plane of the plasma membrane (Li et al., 2012). Currently, the involvement of motor proteins in this process is still very doubtful. Some evidence suggests that kinesins might play a role at different levels. The FRA1 gene encodes a kinesin-4, which was identified by a mutation that induces disorganization in the structure of cellulose by altering the orientation of cellulose microfibrils and therefore by making the cell wall more fragile (Zhong et al., 2002). The protein Fra1 is an authentic kinesin since it moves progressively along microtubules and retains ATPase activity stimulated by microtubules (Zhu and Dixit, 2011b). The role of this kinesin during cellulose deposition is nevertheless unclear. As a true motor, Fra1 could transport cargoes in the cortex of plant cells (where it was localized); although the nature of such cargoes is unknown, an intriguing hypothesis is that Fra1 carries CesA particles to the insertion points in the plasma membrane. Alternatively, Fra1 can regulate the dynamic organization of microtubules in the cell cortex, either allowing them to align properly or fine-tuning their direction below the plasma membrane, two important events for the synthesis and orientation of cellulose microfibrils (Chan, 2012). Brittle Culm12 (*BC12*) is a gene that encodes for a kinesin-4 protein. Like Fra1, bc12 mutants show a significant alteration in the orientation of cellulose microfibrils. Even BC12 is an authentic motor protein but unlike Fra1 is also present in the nucleus and may function as a transcriptional activator (Li et al., 2011). On the basis of flow cytometric analysis and expression assays, BC12 is a kinesin involved in both cell-cycle progression and cellulose deposition (Mu et al., 2010) thus suggesting microtubule-associated proteins as a possible link between cell cycle and cell wall deposition.

If kinesins participate in the assembly of the cell wall, where is their contribution necessary? The movement of CesA in the plane of the plasma membrane is definitely independent of a kinesin-based motor process because CesA can move even in the absence of microtubules. In addition, it is likely that microtubules could direct the movement of CesA, but they do not participate in the making of CesA trafficking. Therefore, kinesins may participate in other steps of the cell wall assembly process, especially during the transport of CesA to the insertion points. We cannot exclude that kinesins may also participate in the assembly of the protein complex that makes CesA as active but the few evidence collected so far suggest that kinesins might determine the insertion of CesA in the plasma membrane. If both Fra1 and BC12 are true motors, it is possible that they carry cargoes containing CesA to the insertion points. Recent data helped to clarify the specific cargo carrying CesA toward the plasma membrane. A particular type of membrane compartment has been identified as critical in either transport or recycling of CesA, the so-called MASC (Crowell et al., 2009) or SmaCC (Gutierrez et al., 2009). This membrane structure derives from the Golgi apparatus and mediates the delivery of CesA from Golgi stacks to the plasma membrane. The precise role of MASC/SmaCC is not clear but it is expected to be a kind of reservoir for CesA before it is inserted into the plasma membrane. The MASC/SmaCC interacts with microtubules because the insertion of CesA occurs only in the presence of intact microtubules. As soon as MASC/SmaCC interacts with microtubules, this compartment may move along microtubules to the insertion point. Experiments with inhibitors suggested that the movement of MASC/SmaCC along microtubules is dependent on the process of polymerization/depolymerization of microtubules (treadmilling) rather than on active movements (Crowell et al., 2009; Gutierrez et al., 2009). However, the protein component of the putative linker has not yet been identified and we cannot exclude a priori that kinesins can be part. So far, no study has correlated either BC12 or Fra1 to the distribution of CesA. Alternatively, BC12 and Fra1 could be involved in the assembly of cellulose microfibrils. In this model, actively moving kinesins might be used to localize in the plasma membrane specific (protein?) components required to organize properly the nascent cellulose microfibrils. Although this hypothesis is entirely speculative, evidence that the local properties of pectin is important for either deposition or orientation of cellulose microfibrils parallel to cortical microtubules might suggest a link between transport of vesicular components and cellulose orientation (Yoneda et al., 2010).

**FIGURE 3:**
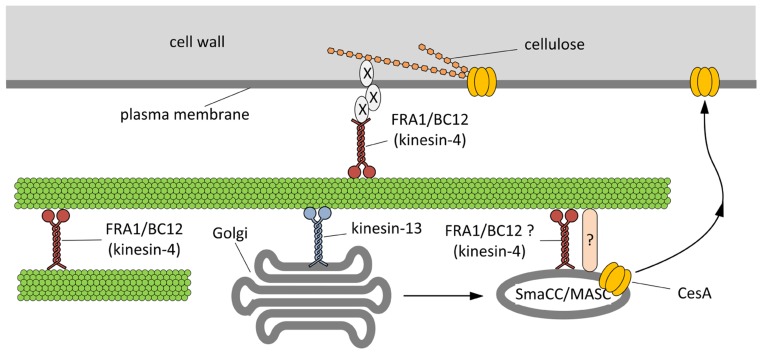
** Hypothetical model outlining the functions of kinesins during the assembly of cell wall.**On the basis of current literature, members of the kinesin-4 subfamily (FRA1 and/or BC12) might be used to organize microtubules beneath the plasma membrane in order to favor either the proper insertion or the activation of cellulose synthase (CesA). The interplay between kinesins and CesA might be also more direct. Members of the kinesin-13 subfamily have been hypothesized either to transport locally or to pause Golgi stacks along microtubules. Once assembled in the Golgi stacks, CesA might move into the so-called SmaCC/MASC compartments, which are known to interact with microtubules, a step required for the insertion of CesA into the plasma membrane. The proteins mediating the interaction between SmaCC/MASC and microtubules are partially known (indicated by question mark) and FRA1/BC12 might putatively be part of such complex. As a further hypothesis, FRA1 and BC12 might be part of the complex that organize the nascent cellulose microfibrils at the plasma membrane interface by delivering specific components that regulate the orientation of cellulose microfibrils (indicated by X).

If the transport of either CesA or MASC/SmaCC does not involve the presence of kinesins, the activity of these motor proteins might be required for a previous step. As described above, members of the kinesin-13 family are most likely involved in regulating the position of Golgi bodies in plant cells (Lu et al., 2005; Wei et al., 2009). Since the pausing of Golgi is characteristic of the process of cellulose synthesis (Crowell et al., 2009), a plausible hypothesis is that microtubules and kinesin-13 members are important in positioning the Golgi bodies to critical points where insertion of CesA in the plasma membrane must occur. Hypothetically, the pausing step might precede insertion of CesA in the membrane as mediated by either MASC/SmaCC or the Trans-Golgi Network (TGN). Kinesins-13 may therefore regulate the insertion of CesA contributing to the synthesis of cellulose (**Figure 3**). The importance of motor proteins during cell elongation is also documented by the microtubule-based movement of the endoplasmic reticulum in characean algae at a stage coinciding with the onset of cell elongation (Foissner et al., 2009). Although the precise relationship between such movement and cellulose organization is unknown, microtubules and microtubule-based motors might be required for locally providing molecules necessary for the early stages of cell growth (which may include synthesis and/or organization of cellulose microfibrils).

Is there evidence that the microtubule-based transport can serve to pause specific organelle classes in plant cells? Secretory vesicles (presumably produced by Golgi) isolated from tobacco pollen tubes can move along microtubules so slowly that are incompatible with cytoplasmic streaming (Romagnoli et al., 2007). These vesicles can also temporarily stop along microtubules and then either resume their movement or detach from microtubules and move along actin filaments. This evidence suggests that secretory vesicles have both motors (kinesin and myosin) on their surface and that microtubule-based motors can be used for either stopping or slowing down membrane structures. Pausing of plant organelles along microtubules seem to be a common trait because it is reported to occur for RNA granules, Golgi bodies, peroxisomes, and mitochondria. All these compartments pause at microtubules but the frequency of pauses is not affected by microtubules depolymerization (Hamada et al., 2012). The authors suggested that pausing on microtubules facilitates the interactions between more stationary organelles (such as ER) and translocating organelles in the cell cortex.

## IS THE MODEL OF FUNCTIONAL COOPERATION EFFECTIVE IN PLANT CELLS?

Assuming that the myosin-based transport might move organelles for longer distances and that kinesins are conversely involved in the fine-tuning, how is the trafficking of plant organelles regulated by the kinesin-myosin interplay? In animal cells, kinesins (and dyneins) direct the movement of organelles along microtubules while myosins often counteract this activity in order to position organelles precisely (Pathak et al., 2010). In this context, kinesins and dyneins work as conventional motors that drive cargoes along predefined pathways. However, organelle transport in plant cells is changed as organelles preferably move along actin cables. Consequently, kinesins might be putatively involved in unconventional motor-driven movements. According to current hypothesis (Muresan and Muresan, 2012), kinesins might have complementary function during organelle movement in plant cells, which may fit relatively with the model of functional cooperation proposed above. As a first hypothesis, microtubules and microtubule-based motors might be used to either counteract or slow-down the activity of myosins in order to regulate precisely the localization of organelles in distinct cell areas. In this case, kinesins and myosins bind to the organelle surface but, while myosin supports the long-range transport of organelles along actin cables, kinesins might be used for a short-range transport or for the local positioning of organelles. This hypothesis is supported by the *in vitro* analysis of organelle/vesicle movement along both actin filaments and microtubules (Romagnoli et al., 2007) and by analysis of microtubule inhibitors on the movement of Golgi bodies (Nebenführ et al., 1999). As supposed for animal cells, kinesins might also transport organelles in an unconventional way by moving short microtubules, to which organelles are attached by non-motor proteins, along stationary microtubules (Muresan and Muresan, 2008). In this case, kinesins are not physically attached to moving organelles but they act like bridges between adjacent microtubules. Currently, no data from plant cells support realistically this hypothesis. Alternatively, kinesins might transport organelles or vesicles by moving short microtubules along stationary actin cables. In such case, organelles are attached to microtubules by non-motor proteins while calponin-domain kinesins are likely to be required for moving microtubules along stationary actin cables. This model recalls the role of the dynein/dynactin complex in animal cells, which is used to move microtubule-anchored vesicles along actin cables in nerve cells (Dillman et al., 1996). In plant cells, the presence of calponin-domain kinesins suggests that the relationships between actin filaments and microtubules might be also regulated by motor proteins (Frey et al., 2009). Although such interactions have been postulated to mediate mainly the relative distribution of actin filaments and microtubules (Preuss et al., 2004), we cannot exclude that calponin-domain kinesins might also be used to redistribute microtubule-associated organelles along actin filaments. Alternatively, the CH domain of kinesins might also be used to interface the organization of actin filaments with the regulation of kinesin moving along microtubules and thus to control organelle/vesicle transport (Umezu et al., 2011). Although experimental evidences of such regulatory mechanism have not been described in plant cells, regulation of kinesin activity by an actin-based trapping process has been recently documented to occur in interphase and mitotic tobacco BY-2 cells. In this case, a kinesin with a calponin homology domain (NtKCH) may exist in two distinct states (dynamic or static) in function of its binding to actin filaments (Klotz and Nick, 2012). Although a specific role for these kinesins has not been yet established, kinesins with a CH domain might link actin filaments and microtubules during nuclear positioning (Frey et al., 2010).

## CONCLUSION

Before the discovery of kinesins, organelle trafficking in plant cells was essentially assumed dependent on actin filaments and myosins (Condeelis, 1974; Williamson, 1979). Now, we are realizing that the fine-tuning of organelle transport also requires the activity of microtubule-based motors. Apparently, the primary role of kinesins is to support the transport activity accomplished by the dynamic interplay between actin filaments and myosins. Although we have a detailed view of the kinesin genes present in some plant species, we still need to determine several aspects of the kinesin-based movement of plant organelles, among which we cite only a couple of examples. The identification of kinesin receptors, such as (putatively) kinectin (Kumar et al., 1995), will allow to determine the specificity of kinesins with respect to their cargoes. Understanding the regulation of kinesin activity is also essential to recognize how the microtubule-based motility is synchronized with the actin-based motility. In this context, it is of particular interest the interaction of Rab proteins with myosins (Seabra and Coudrier, 2004) and with microtubule-based motors (Horgan and McCaffrey, 2011). Either Rab-GTPases might function as “true” receptors for anchoring motors to the organelle surface or as regulators of motor activity; consequently, they might be used to synchronize the activity of both myosin and kinesin and to control precisely the trafficking of plant organelles.

## Conflict of Interest Statement

The authors declare that the research was conducted in the absence of any commercial or financial relationships that could be construed as a potential conflict of interest.
